# The Arg/N-Degron Pathway—A Potential Running Back in Fine-Tuning the Inflammatory Response?

**DOI:** 10.3390/biom10060903

**Published:** 2020-06-14

**Authors:** Dominique Leboeuf, Maxim Pyatkov, Timofei S. Zatsepin, Konstantin Piatkov

**Affiliations:** 1 Skolkovo Institute of Science and Technology, 121205 Moscow, Russia; dominique.leboeuf@skoltech.ru (D.L.); t.zatsepin@skoltech.ru (T.S.Z.); 2 Institute of Mathematical Problems of Biology, Keldysh Institute of Applied Mathematics, Russian Academy of Sciences, Pushchino, 142290 Moscow, Russia; mpyatkov@impb.ru

**Keywords:** Arg/N-degron pathway, UBR-ubiquitin ligases, ubiquitin, proteolysis, inflammatory caspases, inflammation

## Abstract

Recognition of danger signals by a cell initiates a powerful cascade of events generally leading to inflammation. Inflammatory caspases and several other proteases become activated and subsequently cleave their target proinflammatory mediators. The irreversible nature of this process implies that the newly generated proinflammatory fragments need to be sequestered, inhibited, or degraded in order to cancel the proinflammatory program or prevent chronic inflammation. The Arg/N-degron pathway is a ubiquitin-dependent proteolytic pathway that specifically degrades protein fragments bearing N-degrons, or destabilizing residues, which are recognized by the E3 ligases of the pathway. Here, we report that the Arg/N-degron pathway selectively degrades a number of proinflammatory fragments, including some activated inflammatory caspases, contributing in tuning inflammatory processes. Partial ablation of the Arg/N-degron pathway greatly increases IL-1β secretion, indicating the importance of this ubiquitous pathway in the initiation and resolution of inflammation. Thus, we propose a model wherein the Arg/N-degron pathway participates in the control of inflammation in two ways: in the generation of inflammatory signals by the degradation of inhibitory anti-inflammatory domains and as an “off switch” for inflammatory responses through the selective degradation of proinflammatory fragments.

## 1. Introduction

Recognition of a danger signal triggers a coordinated cascade of events, initiating the inflammatory response. In cells of the innate immune system such as neutrophils, granulocytes, or macrophages, danger signals lead to activation of inflammatory caspases (caspases-1, -4, -5, -11, and -12) [1,2] and subsequent proteolysis of a multitude of proinflammatory targets, including IL-1β and various activators of inflammation [3,4]. In the adaptive immune system, cytotoxic T lymphocytes (CTL) and natural killer (NK) cells influence proinflammatory cytokine secretion through the action of granzymes [5,6]. In a more general manner, inflammation can also be initiated through activation of the NF-κB pathway; triggering the transcription of numerous proinflammatory genes; and inducing the production of cytokines, chemokines, and additional proinflammatory mediators [7]. Proinflammatory fragments generated by inflammatory caspases are irreversibly modified, and only sequestration or degradation of the fragments will lead to the resolution of the inflammatory response.

Ubiquitylation is known to regulate many inflammatory pathways such as those mediated by Toll-like receptors, NOD-like receptors, and cytokine receptors, all of which can initiate the NF-κB pathway, which is itself being tightly controlled by adding or removing ubiquitin chains [8,9]. For instance, the processing of NF-κB precursors, activation of the IκB kinase (IKK) complex, and degradation of the NF-κB inhibitor IκB all require ubiquitylation [10,11]. More specifically, when cleaved by calpains during sepsis or other inflammatory settings [12], the NF-κB inhibitor IκBα can be degraded by the Arg/N-degron pathway, due to the presence of the destabilizing residue Glu at the newly exposed N-terminus [13]. The negative regulation of inflammation is generally provided by deubiquitinating enzymes, which remove the ubiquitin-activating signal [8,14]. However, recent evidence suggests that the resolution of inflammation could also be mediated by E3 ligases and protein degradation through Lys48-linked ubiquitin chains [15,16,17]. An example is the control of cytokine signaling provided by the E3 ligases Suppressor of Cytokine (SOC) signaling proteins, which are themselves regulated by ubiquitin-mediated degradation [18].

**Figure 1:**
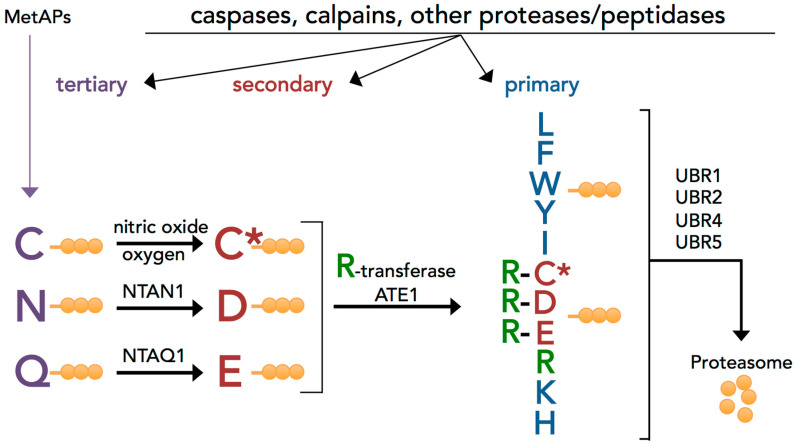
The mammalian Arg/N-degron pathway. N-terminal residues are indicated by single-letter abbreviations for amino acids, and the rest of a protein substrate is represented by orange spheres. “Primary”, “secondary”, and “tertiary” denote mechanistically distinct subsets of destabilizing N-terminal residues. C* denotes oxidized N-terminal Cys, either Cys-sulfinate or -sulfonate. The mammalian N-recognins UBR1, UBR2, UBR4, and UBR5 (EDD) have multiple substrate-binding sites that recognize internal and terminal N-degrons and target them to the proteasome. MetAPs: methionine aminopeptidase, NTAN1: N-terminal asparagine amidohydrolase, and NTAQ1: N-terminal glutamine amidohydrolase (adapted from [21]).

The N-degron pathway is a proteolytic pathway that relates the in vivo half-life of a protein to the identity of its N-terminal residue [19]. N-degrons comprise a destabilizing residue, which can be recognized by an E3 ubiquitin ligase, as well as an internal lysine that acts as the polyubiquitylation site. N-degrons, generally formed after the cleavage of proteins by exo- or endopeptidases [13,20,21,22], are recognized by specific E3 ubiquitin ligases (N-recognins) that target the proteins for degradation through the proteasome or autophagy [23,24]. In mammals, these E3 ligases are UBR1, UBR2, UBR4, and UBR5 [25]. The eukaryote N-degron pathway consists of five branches [19], including the Arg/N-degron pathway, which targets specific nonacetylated N-terminal residues (Figure 1) [26]. The primary destabilizing N-terminal residues are directly recognized by E3 ligases, whereas N-terminal Asp, Glu, Asn, Gln, and Cys amino acids function as destabilizing residues through their preliminary modifications, such as N-terminal arginylation by the ATE1 arginyl-transferase (R-transferase) (Figure 1) [26,27].

Through the selective degradation of proteins, the mammalian Arg/N-degron pathway mediates multiple biological functions, including the regulation of apoptosis, repression of neurodegeneration, the elimination of aberrant proteins during stress, as well as the regulation of vascular development and cell motility ([19] and references therein). The regulation of apoptosis by the Arg/N-degron pathway involves the selective degradation of caspase-cleaved proapoptotic fragments that contain N-degrons [21]. In a similar manner to that of apoptosis, the onset of inflammation results in cleavage by activated proteases of multiple proteins, which could also be potential Arg/N-degron substrates. Recently, a role for the Arg/N-degron pathway in the development of the immune response against anthrax lethal factor (LF) was discovered. Upon infection, the LF protease cleaves the mouse inflammasome NLRP1B and generates a destabilized neo-N terminus that is recognized by the UBR2 E3 ligase of the Arg/N-degron pathway. Degradation of the N-terminal fragment frees the CARD domain of the NLRP1B, which can then recruit caspase-1 and initiate pyroptosis [28,29]. These studies were the first to indicate a role for N-terminal degradation in the onset of an inflammatory response. Here, we propose a broader mechanism where the Arg/N-degron pathway can participate in the control of inflammation by degrading proinflammatory fragments, thus protecting cells from the misfiring of inflammatory caspases and contributing in the resolution of the inflammatory state.

Thus, we suggest that the Arg/N-degron pathway actively participates in the regulation of inflammation in a dual manner: through the selective degradation of inhibitory anti-inflammatory domains and a number of proinflammatory fragments, including some activated inflammatory caspases, contributing in tuning inflammatory processes.

## 2. Materials and Methods

### 2.1. Bioinformatic Analysis of Caspase Substrates

Human inflammation caspase cleavage sites were collected from the MEROPS database [30]. The search for orthologous sites was produced using pBLAST [31] with an e-value cutoff of 1 × 10^−16^. The database subset was obtained from a nonredundant database restricted by the taxon Vertebrates (taxon id: 7742). Input sequences for pBLAST, including human octamer (P4-P4’) +/−26 surrounding amino acids, 60 amino acids in total, were retrieved from the Uniprot database [32]. Subsequent data analysis was performed using an in-house package R language [33].

### 2.2. Cell Culture, Transfections, and Stimulations

The mouse J774A.1 macrophage cell line (ATCC^®^ Manassas, VA, USA, TIB-67^TM^) was grown in DMEM supplemented with 10% FBS (Gibco, Waltham, MA, USA, #161400) and 2-mM L-glutamine (Gibco, #250300) without antibiotics. Cells were split when they reached 80% confluency. siRNAs were transfected using Lipofectamine RNAiMAX (Invitrogen, Waltham, MA, USA, #13778), according to the manufacturer’s instructions, at a total of 5 or 10 nM (1.25 or 2.5 nM for each UBR-ubiquitin ligase siRNA). Cells were stimulated with LPS (O11:B4, Millipore Sigma St. Louis, MO, USA, #L4391) at 1, 10, or 50 ng/mL for 6 h or 24 h, with ATP (5 mM) added during the last hour or with staurosporine at 500 nM for 24 h (Millipore Sigma, St. Louis, MO, USA, #S6942) in DMEM with 2% FBS.

### 2.3. Plasmids, cDNAs, and Primers

DH5α Escherichia coli strain (Invitrogen, Waltham, MA, USA, #EC0111) was used for the cloning and production of plasmids. Phusion High-Fidelity DNA polymerase (New England Biolabs, Ipswich, MA, USA, #M0530) was used for PCR. Sequences of all constructed plasmids were verified by Sanger sequencing. The plasmid pKP496 was constructed by the ligation of annealed primers 1447 and 1448 into SacII/XbaI-cut pcDNA3^f^DHFR Ub^R48^Xpr [34]. The resulting plasmid, which encoded the ^f^DHFR-Ub^K48R^-MCS (SacII-EcoRI-XhoI-ClaI-EcoRV)-flag fusion, was used to construct the plasmids used in this study for the ubiquitin reference technique [35] (Table S2 and descriptions). All plasmids generated in this study are available from the lead contact upon request.

### 2.4. In Vitro Transcription–Translation–Degradation Assay

The TNT T7-Coupled Transcription/Translation System, (Promega, Madison, WI, USA, #L1170) was used to carry out transcription–translation–degradation assays. Reaction samples were prepared according to the manufacturer’s instructions. Newly formed proteins in reticulocyte extract were pulse-labeled with l-[^35^S]methionine (0.55 mCi/mL, 1′000 Ci/mmol; MP Biomedicals, Solon, OH, USA) for 5 min in a total volume of 30 μL. The labeling was quenched by the addition of cycloheximide and unlabeled methionine to the final concentrations of 0.1 mg/mL and 5 mM, respectively. Unless stated otherwise, the reactions were carried out at 30 °C and terminated by the addition of an equal volume of TDS (tris-dodecylsulfate) buffer (1% SDS, 5-mM DTT, 50-mM Tris·HCl, pH 7.4, also containing a complete protease inhibitor mixture; Roche, Indianapolis, IN, USA, 5892791001) followed by heating at 95 °C for 10 min. The resulting samples were diluted with 10 volumes of TNN (tris-nonidet-NaCl) buffer (0.5% nonidet P-40, 0.25-M NaCl, 5-mM EDTA, 50-mM Tris·HCl, pH 7.4, also containing the complete protease-inhibitor mixture; Roche, Indianapolis, IN, USA), and the amounts of ^35^S were measured by precipitating an aliquot with 10% trichloroacetic acid, followed by counting in a liquid scintillation counter (Beckman Coulter, Brea, CA, USA, LS6000). For immunoprecipitation, samples were adjusted to contain equal amounts of total ^35^S and were added to 10-μL beads with an immobilized antibody, anti-FLAG M2 (F1804; Sigma, St. Louis, MO, USA). The samples were incubated with rocking at 4 °C for 4 h, followed by four washes with TNN buffer, resuspension in a 20-μL SDS sample buffer, and heating at 95 °C for 10 min, followed by SDS 4–15% PAGE and autoradiography. Quantification of autoradiograms was carried out using PhosphorImager and ImageQuant 5.0 software (Molecular Dynamics, Chatsworth, CA, USA).

### 2.5. siRNA Description and Selection

siRNAs targeting mouse UBR1, UBR2, UBR4, or UBR5 (NCBI Genbank accession codes NM_009461.2, NM_001177374.1, NM_001160319.1, and NM_001081359.3) were designed with the lowest off-target potential, including miRNA-like activity and decreased capacity to activate innate immunity. Screening and selection of the most efficient and potent siRNA (Table S4) are described in [36]. The control siRNA targets the Firefly Luciferase gene.

### 2.6. cDNA Synthesis and qPCR

Total RNA was purified by disrupting cells in Trizol (Invitrogen, Waltham, MA, #155960) using the MP FastPrep-24 instrument and Lysing Matrix D (MP Biomedicals, Solon, OH, USA, SKU 116913050-CF), followed by precipitation with isopropanol according to the manufacturer’s instructions. cDNA was generated using the High-Capacity cDNA Reverse Transcription Kit (Applied Biosystems, Waltham, MA, USA, #43688). Levels of mRNA were assessed by quantitative PCR using SYBR Green (Thermo Fisher, Waltham, MA, USA, 4364346) in the QuantStudio 5 thermocycler (Applied Biosystems Waltham, MA, USA). The mRNA levels were normalized to the level of the housekeeping gene (GAPDH) and to the average value of the control group. Specific primers used in qPCR are listed in Table S3.

### 2.7. Cell Extracts and Western Blot

Cells were lysed in RIPA buffer containing the complete protease inhibitor cocktail (Roche) using the MP FastPrep-24 instrument and Lysing Matrix D (MP Biomedicals, Solon, OH, USA). The extracts were centrifuged at 12,000× *g* for 10 min at 4 °C. Media samples were concentrated ~6× using ultracentrifugation filtration units with a MWCO of 3kDa (Amicon, St. Louis, MO, USA, UFC8003). Total protein concentrations in the lysates and supernatants were determined using the BCA assay (Pierce, Waltham, MA, USA, #2322). Samples were diluted in LDS sample buffer (ThermoFisher, Waltham, MA, USA, #NP0008) supplemented with 25-mM DTT and heated at 95 °C for 10 min, except for the detection of UBR4, where samples were heated at 56 °C for 30 min. Protein analysis was performed in SDS 5–12% PAGE, with 10–100 μg of total protein loaded per lane. PAGE-fractionated proteins were transferred onto the nitrocellulose membrane and analyzed by Western blot using the following antibodies: anti-UBR1 (Abcam, Cambridge, MA, USA, #156436), anti-UBR2 (Abcam #191505), anti-UBR4 (Abcam #86738), anti-EDD (Santa Cruz, Dallas, TX, USA, #515494), anti-GAPDH (Santa Cruz #sc32233), anti- IL-1β (Cell Signaling, Danvers, MA, USA, #12507), anticleaved-caspase-1 (P20) (Cell Signaling #4199), and anti-caspase-3 (Cell Signaling #14220). Immunoblots were visualized using the SuperSignal West Femto reagent (Pierce, Waltham, MA, USA, #34095) according to the manufacturer’s instructions in the Fusion Solo S Imager (Vilber Lourmat, Marne-la-Vallée, France).

### 2.8. Caspase-1 Activity Assay

Caspase-1 activity was measured using the bioluminescent Caspase-Glo 1 Inflammasome Assay (Promega, Madison, WI, USA) in cell supernatants from J774A.1 cells stimulated with 100-ng/mL LPS or 500-nM staurosporine for 24 h following the manufacturer’s instructions, using Ac-YVAD-CHO as a specific caspase-1 inhibitor.

### 2.9. Cytokine Bead Assay (CBA)

Cytokine levels were measured by the cytokine bead assay (BD Bioscience, Franklin Lakes, NJ, USA, Flex Set #560232) according to the manufacturer’s instructions. Briefly, cell culture media was concentrated 5-6x using ultracentrifugation filtration units with a MWCO of 3 kDa (Amicon, UFC8003) following the manufacturer’s instructions. Total protein concentrations in the supernatants were determined using the BCA assay, and 160 µg of protein was assessed in each sample. Data was acquired using a BD LSRFortessa^TM^, and analysis of flow cytometry data was performed using FlowJo software, version 10.4.1 (BD Bioscience).

### 2.10. Statistical Analysis

Prism 7 (GraphPad Software, La Jolla, CA, USA) was used for statistical analyses. A one-way ANOVA was used for statistical analysis unless otherwise indicated. A *p* < 0.01 was considered significant, unless otherwise indicated. 

## 3. Results

### 3.1. Evolutionary Conserved Proinflammatory Fragments Contain Destabilizing Residues at Their N-Terminus

**Figure 2:**
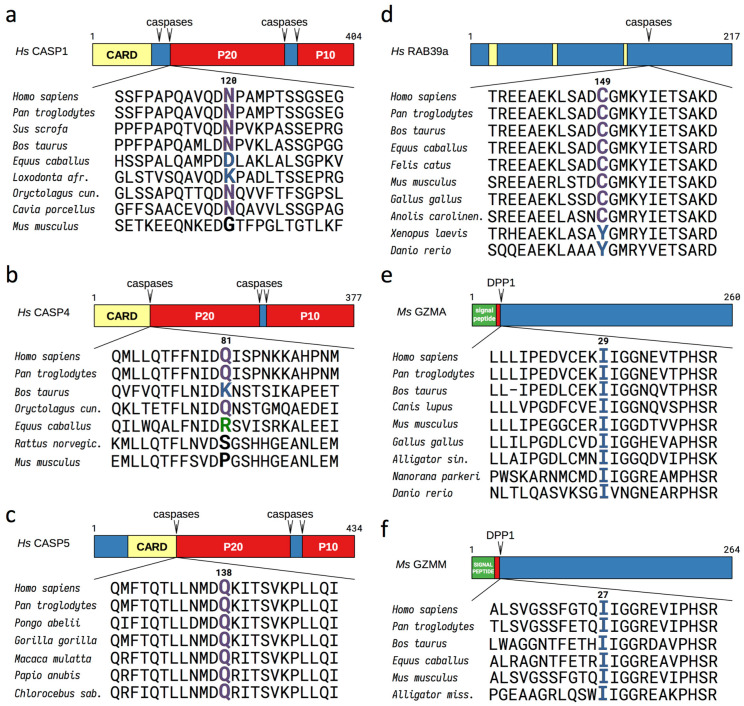
Evolutionary conservation of the cleavage sites and destabilizing P1′ residues in the proinflammatory protein fragments Asn-CASP1 (**a**), Gln-CASP4 (**b**), Gln-CASP5 (**c**), Cys-RAB39a (**d**), Ile-GRZA (**e**), and Ile-GRZM (**f**). These fragments have been shown, in the present work, to be short-lived substrates of the Arg/N-degron pathway (Figure 3). These proapoptotic fragments are generated by autoprocessing, by caspases, or by other endopeptidases. Cleavage sites are indicated by arrowheads. The indicated residue numbers, including the numbers of the P1′ residues (larger letters), are of the human or mouse versions of the cited proteins. Approximate locations and names of specific protein domains are indicated as well. These fragments are described in the Supplementary Results.

The initiation and progression of inflammation involves the activation of specific proteases that drive and amplify inflammatory signaling by cleaving their protein targets. We reasoned that some protein fragments resulting from processing by activated inflammatory caspases could be potential Arg/N-degron substrates. A search of online databases for human and orthologous proteins containing caspase-1 cleavage sites revealed more than 120 known substrates, 21% of which bear a destabilizing residue according to the Arg/N-degron pathway at their P1′ position (the first residue after the cleavage site) (Table S1). From the shorter list of experimentally confirmed caspase-1 substrates with inflammatory functions, we identified nine fragments with possible N-degrons: Asn^120^-CASP1, Gln^81^-CASP4, Gln^138^-CASP5, Cys^149^Rab39a, Tyr^37^-IL-18, Tyr^49^-CCL3, Glu^245^ and Leu^249^-Ataxin-3, Cys^50^-hnRNPA2 (heterogeneous nuclear ribonucleoproteins A2/B1), and Leu^680^-Matrin-3 (the fragments are summarized in Table S1). Interestingly, all of the destabilizing N-terminal residues studied are conserved through evolution, with few exceptions (Figure 2 and Figure S1). These proinflammatory fragments are further described in the SI Results.

The inclusion of a destabilizing residue after the self-cleavage site in caspase-1, -4, and -5 is conserved in most mammals in which this cleavage motif is present (Figure 2). However, in rodents, even if caspase-1 and caspase-11 contain self-cleavage sites, the P1′ residue is not recognized by the Arg/N-degron pathway, indicating a possible evolutionary transition towards a degradable inflammatory caspase fragment in higher mammals. The degradation of activated caspase-1 and -11 in rodents is most likely driven by other mechanisms. In all other caspase-generated proinflammatory fragments examined in this study, the destabilizing residue at the N-terminus is well-conserved throughout vertebrates (Figure 2 and Figure S1), with few exceptions. Tellingly, however, all of the changes in the P1′ residues remain destabilizing in the Arg/N-degron pathway. Knowing that more than 80% of the mapped caspase-cleavage sites in cellular proteins contain small residues such as Gly, Ser, Thr, and Ala at their P1′ positions [37], residues that are not recognized by the Arg/N-degron pathway, the change for a destabilizing residue in these proinflammatory fragments in higher mammals is significant and could indicate a fitness-increasing property that was later maintained by selection during evolution.

Proinflammatory fragments containing destabilizing residues at their N-terminus are also generated by other endopeptidases such as DPP1 and proteinase-3. These fragments include Ile^29^-GRZA, Ile^27^-GRZM, Ile^26^-GRZK, Glu^6^-IL-36β, and Tyr^16^-IL36γ (Figure 2 and Figure S1). The N-terminal destabilizing residues present in granzymes A, M, K, and IL36 are conserved in all organisms examined, illustrating the evolutionary pressure to keep the degradation signal and demonstrating the significance of the N-degron pathway in the regulation of these fragments. A detailed description of the proinflammatory fragments with possible N-degrons listed there is available in the Supplementary Materials.

### 3.2. Proinflammatory Fragments Generated by Proteases are Targeted for Degradation by the Arg/N-Degron Pathway

**Figure 3:**
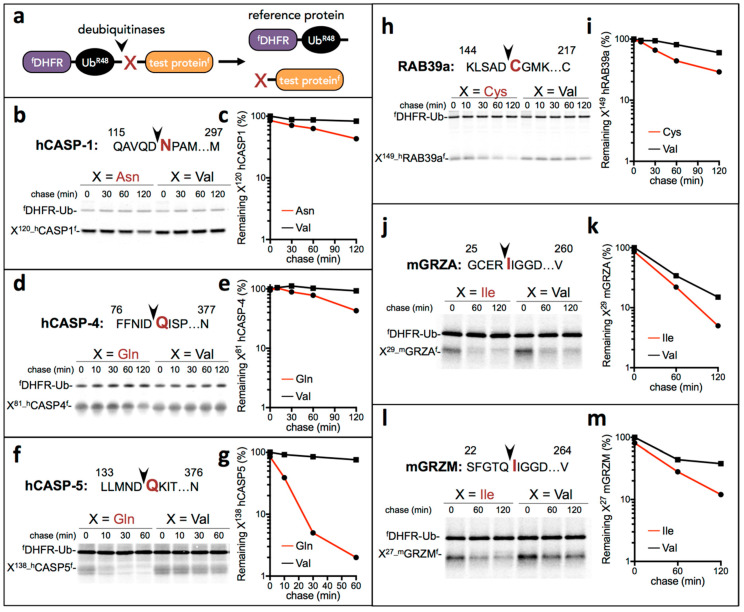
Proinflammatory fragments are short-lived N-degron pathway substrates. (**a**) The URT assay [21,38]. (**b**) The cleavage site in human CASPASE-1 is indicated by an arrowhead, with the destabilizing residue shown in red. Residue numbers are the numbers of the first-shown residue and the last residue of the full-length protein. Asn^120^-hCASP-1 (produced from ^f^DHFR-Ub^R48^-Asn^120^-hCASP-1^f^) and Val^120^-hCASP-1 were expressed in the reticulocyte extract and labeled with ^35^S-Met/Cys for 10 min at 30 °C, followed by a chase, immunoprecipitation with an anti-flag antibody, SDS/PAGE, and autoradiography. (**c**) Quantification of B using the reference protein ^f^DHFR-Ub^R48^. (**d**) Same as b but with human X^81^-CASP-4^f^ (X = Gln and Val). (**e**) Quantification of d. (**f**) Same as b but with human X^138^-CASP-5^f^ (X = Gln and Val). (**g**) Quantification of f. (**h**) Same as b but with human X^144^-RAB39a^f^ (X = Cys and Val). (**i**) Quantification of h. (**j**) Same as b but with mouse X^29^-GRZA (X = Ile and Val). (**k**) Quantification of j. (**l**) Same as b but with mouse X^27^-GRZM (X = Ile and Val). (**m**) Quantification of l.

To determine whether proinflammatory fragments such as caspase-1, -4, and -5 are degraded by the Arg/N-degron pathway, we used the Ubiquitin reference technique (URT) [21,38] where the test protein is fused to a reference ^f^DHFR-Ub^R48^, a FLAG-tagged derivative of the mouse dihydrofolate reductase (Figure 3a). Cotranslational cleavage of the fusion protein by deubiquitylases produces both the test protein and the reference polypeptide at an initial equimolar ratio. Relative degradation rates of the test protein can be quantified by normalization to the level of the ^f^DHFR stable reference at the same time point.

We first examined inflammatory caspases for degradation by the Arg/N-degron pathway. Self-cleavage of caspase-1 after the CARD domain is necessary to generate a more stable active form of the proteolytic enzyme and even serves to terminate inflammasome activity [39]. As for human caspases -4 and -5, activity can result of the nonprocessed or the cleaved forms of the enzymes [40,41], while murine caspase-11 autoproteolysis is essential for inflammasome activation [42]. To demonstrate that, after self-cleavage, the remaining C-terminal protein fragments are targets of the Arg/N-degron pathway, specific URT fusions tagged with the flag epitope at the C-terminus were labeled with ^35^S-Met/Cys, followed by a chase, immunoprecipitation with a monoclonal anti-FLAG antibody, SDS/PAGE separation, and quantification by autoradiography (Figure 3b–g). Indeed, the P20-P10 fragments Asn^120^-CASP1, Gln^81^-CASP4, and Gln^138^-CASP5 were degraded quickly in the reticulocyte extract, whereas the otherwise identical fragments bearing the N-terminal Val residue were either stable or nearly stable. These results indicate that the three human inflammatory caspases contain Arg/N-degrons and are targeted for degradation by this pathway. Since caspases are multi-subunit proteins that function as dimers, degradation of one subunit is enough to stop the catalytic activity of the enzyme [43]. Therefore, degradation of the inflammatory caspases by the Arg/N-degron pathway could be an important mechanism to stop the propagation of the inflammation program.

Next, we examined whether proinflammatory fragments generated by inflammatory caspase proteolysis or other proteases contained N-degrons. We confirmed experimentally that the Cys^149^-Rab39a fragment generated after caspase-1-mediated cleavage was indeed a substrate of the Arg/N-degron pathway by using the Ub-reference technique described above (Figure 3h,i). Although cysteine is a tertiary destabilizing residue (Figure 1), it is rapidly oxidized, arginylated, and degraded by the N-degron pathway [27,44]. The oxidation of cysteine requires nitric oxide, which is always present in mammalian cells but increased during inflammation, accelerating the covalent modification of N-terminal Cys [45]. Finally, we examined the degradation of activated granzymes A and M, which are known to increase the production and release of inflammatory cytokines. Processing of the granzyme activation peptide by the protease DPP1 exposes an Ile, which can be recognized by the N-degron pathway. Indeed, both granzyme A and granzyme N were short-lived compared to identical fragments bearing a Val residue at the N-terminus (Figure 3j–m). However, changing the Ile to a Val did not completely stabilize the activated granzymes, suggesting multiple pathways for the degradation of these enzymes.

In sum, we examined 6 of 14 proinflammatory fragments with potential N-degrons (Figure 3) and found that all of them are degraded by the Arg/N-degron pathway. For all fragments except granzymes, the Arg/N-degron pathway is the main mechanism of degradation, at least in reticulocyte extracts.

### 3.3. Partial Downregulation of the Arg/N-Degron Pathway Leads to an Enhanced Inflammatory Response

Since inflammatory caspases and some of their substrates are targets of the Arg/N-degron pathway, even a partial ablation of this pathway should stabilize proinflammatory fragments in the cell and enhance the inflammatory response. Rab39a is a necessary binding partner to caspase-1 for the cleavage and secretion of IL-1β [46,47]. As a result, the most straightforward consequence of the stabilization of caspase-1 or Rab39a would be the increased secretion of IL-1β.

**Figure 4:**
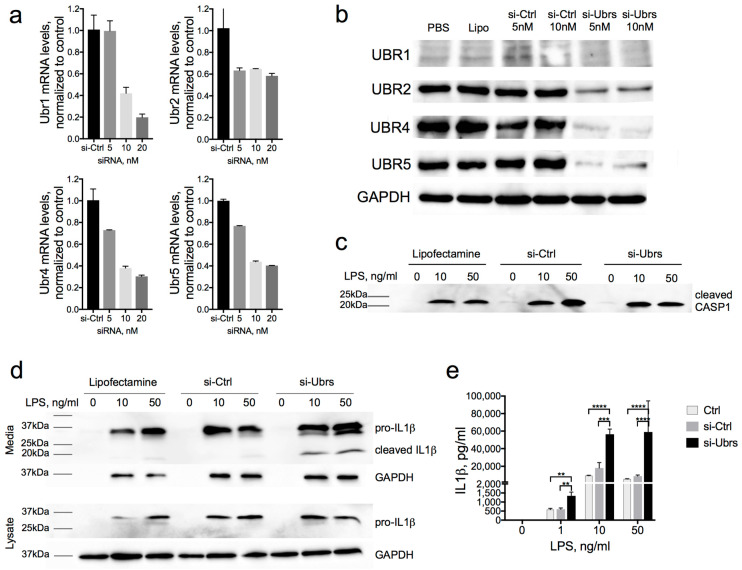
Increased IL1β secretion after the downregulation of Ubr-ubiquitin ligases of the Arg/N-degron pathway. (**a**) mRNA and (**b**) protein levels of UBR1, UBR2, UBR4, and UBR5 in J774A.1 cells after 72 h of exposure to 5 or 10-nM siRNA. (**c**) Western blot analysis of cleaved CASPASE-1 secreted in the media of J774A.1 cells treated with 10 nM of si-Ctrl or siRNA against Ubr (UBR1, UBR2, UBR4, and UBR5) for 72 h, followed by 1, 10, or 50 ng/mL of LPS for 6h and 5-mM ATP for the last hour. (**d**) Western blot analysis of pro- and cleaved IL-1β from the media and lysates of cells treated as in (**c**). (**e**) Quantification of the secreted pro- and cleaved IL-1β from the media of J774A.1 cells treated as in (**c**) by the cytokine bead assay (CBA) *P*-values were determined by a one-way ANOVA (** *p <* 0.01, *** *p <* 0.001 and **** *p <* 0.0001).

We used an RNAi approach to downregulate all four UBR-ubiquitin ligases of the Arg/N-degron pathway in the J774A.1 mouse macrophage cell line. The siRNA used were chemically modified and designed to avoid recognition by TLRs and initiation of an innate immune response [48]. Forty to sixty percent downregulation of mRNA and 50–90% downregulation of the proteins was achieved in J774A.1 cells after 72 h of exposure to siRNA (Figure 4a,b). In a previous work [36], we demonstrated robust UBR1 protein downregulation using the same siRNA. However, because of low levels of UBR1 expression in mouse macrophages, mRNA levels were relied on to confirm the downregulation in this study. To evaluate the levels of IL-1β production in macrophages with downregulated UBR-ubiquitin ligases, we stimulated J774A.1 cells with 1, 10, or 50 ng/mL of LPS for 6 h. Western blot and cytokine bead assay analysis revealed a significant increase in the secretion of IL-1β in the media of cells with a downregulated Arg/N-degron pathway (Figure 4d,e). The assays recognize both pro- and cleaved IL-1β, but the Western blot assay clearly demonstrated the dramatic increase of the cleaved portion of IL-1β in UBR knockdown compared to the control, especially at the lower LPS concentration. Predictably, the amounts of cleaved caspase-1 were not changed in UBR KD macrophages, since the mouse-cleaved caspase-1 is not a substrate of the Arg/N-degron pathway (Figure 4c). Importantly, there was no secretion of IL-1β without LPS stimulation, indicating that the downregulation of UBR-ubiquitin ligases did not induce inflammation in itself. This also indicates that the downregulation of the Arg/N-degron pathway only sensitizes cells to inflammation and requires a proinflammatory signal such as LPS to induce a cytokine response. Together with the URT assays, these results demonstrate that the Arg/N-degron pathway is capable of regulating the level of IL-1β secretion through its ability to degrade proinflammatory fragments.

### 3.4. N-Recognins are not Degraded During the Inflammatory Response

**Figure 5:**
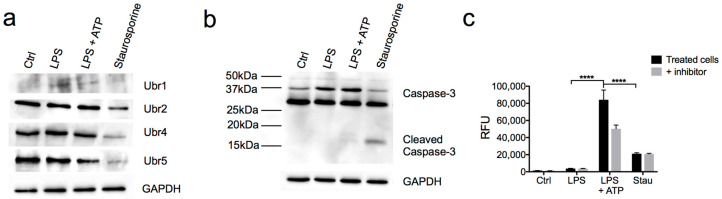
N-recognins of the Arg/N-degron were not degraded during inflammation. (**a**) Western blot analysis of UBR-ub ligases of the Arg/N-degron pathway after caspase-1 or caspase-3 activation. Cells were treated with LPS (100 ng/mL) or staurosporine (500 nM) for 24 h and assessed for the 4 UBR ub-ligases, for caspase-3 (**b**), and for caspase-1 activity (**c**). Caspase-1 activity was measured in the media of cells treated as in (**a**) in presence of the caspase-1-specific inhibitor Ac-YVAD-CHO in half of the wells. *P*-values were determined by a one-way ANOVA (**** *p* < 0.0001).

Apoptotic caspases can cleave N-recognins of the Arg/N-degron pathway during late-apoptosis, once the proapoptotic signaling exceeds the antiapoptotic activity of the Arg/N-degron pathway [21]. Unlike apoptosis, cells can recover from a robust proinflammatory signal and would require a functional Arg/N-degron pathway in order to degrade proinflammatory proteins. Therefore, if the N-degron pathway is instrumental in the resolution of the inflammatory response, then the E3 ligases of the pathway should not get degraded after the activation of inflammatory caspases. To test this hypothesis, we used LPS to generate an inflammatory response and staurosporine to cause apoptosis in a murine macrophage cell line. We examined protein levels of N-recognins of the Arg/N-degron pathway after stimulation with LPS and found that these levels were comparable to controls, while they are significantly decreased in cells treated with staurosporine (Figure 5a). The activation of caspase-1 and caspase-3 was confirmed in the experimental setting (Figure 5b,c). These results indicate that, contrarily to apoptotic conditions, UBR1, UBR2, UBR4, and UBR5 were not cleaved or degraded during inflammation.

## 4. Discussion

Proteolytic processing is a widespread mechanism used by cells to initiate and amplify signaling. One explicit example is the cleavage of multiple proteins by caspases once the apoptotic or inflammatory programs are initiated [49,50]. The irreversible nature of this process implies that activated fragments either need to be sequestered, inhibited, or degraded to turn off the signal. Previous studies demonstrated that the Arg/N-degron pathway contributes to the elimination of proapoptotic signals generated by caspases and calpains at the onset of programmed cell death [13,21,51,52,53]. These proapoptotic fragments are recognized by their newly generated N-terminus following cleavage by proteases. Similar activities occur after danger signals are perceived by cells and inflammatory endopeptidases are activated [54,55]. These newly generated proinflammatory molecules should be inactivated in order to cancel the inflammatory cascade and allow cells to recover from the inflammation. In the present study, we identified in silico fourteen proinflammatory protein fragments with destabilizing N-terminal amino acids, six of which (Asn^120^-CASP1, Gln^81^-CASP4, Gln^137^-CASP5, Cys^149^-Rab39a, Ile^29^-GRZA, and Ile^27^-GRZM) are experimentally shown to contain N-degrons and be substrates of the Arg/N-degron pathway (Figure 3).

Our results illustrate that one of the strategies used by cells to eliminate proteolytically activated fragments generated during inflammation is degradation via their built-in degron, which is exposed upon activation or proteolytic cleavage. Proof of the importance of this mechanism is through the strong evolutionary conservation of the destabilizing nature of N-terminal residues at the P1′ position of the proinflammatory fragments. A constraint of this kind could only be expected if a short in vivo half-life of a proinflammatory fragment was a fitness-increasing property. Additionally, as inflammation and apoptosis most probably originate from a common ancestor molecular pathway, where the evasion of pathogens occurred by “cell suicide” [56,57,58], it would be natural to conserve a common regulatory mechanism as well. However, unlike programmed cell death, cells can recover from the inflammatory process, which could explain why inflammatory caspase-1 does not cleave the E3 ligases of the Arg/N-degron pathway, leaving them intact and able to target proinflammatory fragments for degradation throughout all the stages of inflammation. By destroying proinflammatory activating signals, the Arg/N-degron pathway participates in the resolution of inflammation.

One significant ramification from the suggested function of the Arg/N-degron pathway would be exaggerated inflammatory responses in cells with a malfunctioning Arg/N-degron pathway. Indeed, we found that, when the E3 ligases of this pathway are downregulated, the consequence upon inflammatory stimuli such as LPS is a much greater secretion of IL-1β, even at low concentrations of the endotoxin. In accordance with our findings, partial ablation of the Arg/N-degron pathway in humans and animal models also leads to increased inflammation or inflammation-prone phenotypes. Patients with the Johanson-Blizzard syndrome, who have mutations in the *UBR1* gene leading to a loss-of-function of this protein, exhibit increased inflammatory infiltrates in the pancreas, leading to acinar cell destruction and pancreatic insufficiency [59,60]. *Ubr1*^−/−^ mice are also more sensitive to induced pancreatitis, as shown by increased elastase activity and an elevated systemic inflammatory response upon cerulean dosing [61,62]. In addition, postnatal deletion of the arginyltransferase Ate1 in mice causes brain edema [63], a general indicator of inflammation. Ate1 is essential for the recognition of destabilizing N-terminal residues requiring arginylation (Figure 1), which are present in proinflammatory fragments Cys^149^-Rab39a, Glu^6^-IL36b, and Glu^245^-Ataxin 3, to name a few. Finally, partial inhibition of the Arg/N-degron pathway by the downregulation of UBR1, UBR2, UBR4, and UBR5 in the mouse liver causes the infiltration of neutrophils and eosinophils, known effectors of inflammation [36], and elevates serum IL-1β levels upon LPS stimulation (D. Leboeuf, T. Zatsepin, and K. Piatkov, unpublished data). In sum, these phenotypes indicate that ablation of the Arg/N-degron pathway not only impairs the resolution of inflammation but also increases susceptibility to a stronger inflammatory response, even in the presence of a weak stimulus.

**Figure 6:**
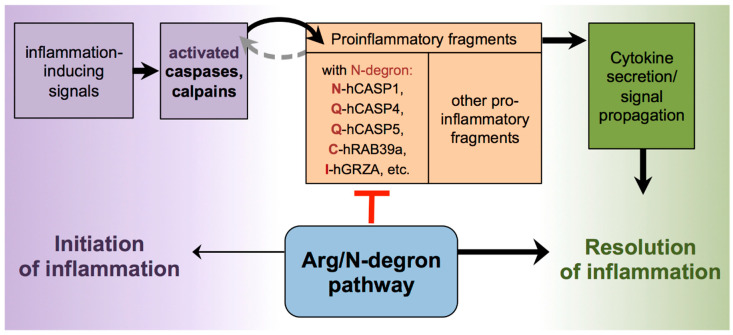
The Arg/N-degron pathway as a regulator of inflammation. Working model where the N-degron pathway regulates inflammation by the degradation of proinflammatory Arg/N-degron substrates (Asn-hCASP-1, Gln-hCASP-4, Gln-hCASP-5, Cys-RAB39a, Ile-GRZA, etc.). Selective degradation of proinflammatory protein fragments by the Arg/N-degron pathway could act as a control mechanism to prevent overstimulation of the immune system and to resolve inflammation.

In light of the results presented in this study as well as recently published data, we propose a model wherein the Arg/N-degron pathway participates in the control of inflammation in two ways: (a) in the generation of inflammatory signals by the degradation of inhibitory anti-inflammatory domains and (b) in the resolution of inflammatory states through the selective degradation of inflammatory mediators or proinflammatory fragments (Figure 6). This dual control mechanism of the immune response by the Arg/N-degron pathway is also known in plants. For instance, the knockout of key components of the Arg/N-degron pathway in *Arabidopsis thaliana* prevents mounting an immune response to a variety of pathogens, which suggests that this pathway is involved in the degradation of proteins involved in the initiation of the immune response [64]. Conversely, the Arg/N-degron pathway is involved in the degradation of protein fragments cleaved by the *Pseudomonas syringae* protease effector AvrRpt2, and the removal of these fragments could help in the resolution of the immune response in the plant [65].

Additional links between the Arg/N-degron pathway and inflammation can be made through its roles in the protein quality control mechanism [66], a cellular process present in all cells from yeast to mammals and, when impaired, is responsible for the generation of inflammation in diseases such as Alzheimer’s and Parkinson’s [67,68,69]. Moreover, due to the ubiquitous expression of the N-degron pathway, inflammation control mechanisms provided by this pathway are present in any cell type, acting as an on/off “switch” for the initiation of an inflammatory response and/or as a “pro-resolving” mediator by means of the degradation of proinflammatory fragments, depending on the cell type and on the source of the stimuli.

## 5. Conclusions

This study provides new evidence that the Arg/N-degron pathway participates in the regulation of inflammation through the targeted degradation of proinflammatory fragments. While inflammation is critical to the response to danger signals and the healing process, it also contributes to the pathophysiology of many noninfectious diseases. Understanding the mechanisms behind the resolution of inflammation is key to the better management of this reaction in many disorders. Our findings propose that the Arg/N-degron pathway has dual functions in the control of inflammatory processes. Ablation of the Arg/N-degron pathway not only impairs the resolution of inflammation but also increases susceptibility to a stronger inflammatory response, even in the presence of a weak stimulus. This feature can be helpful for pharmacological intervention in inflammation, both in the context of cancer treatment and the resolution of chronic inflammation.

## Data Availability

The data that support the findings of this study are available from the corresponding author upon reasonable request.

## Supplementary Materials

The following are available online at https://www.mdpi.com/2218-273X/10/6/903/s1: Table S1. Proinflammatory fragments with destabilizing N-terminal residues. Table S2. Plasmids used in this study. Table S3. Primers used in this study. Table S4. List of siRNA used in this study. Supplemental Methods. Description of the plasmids used in this study. Supplemental Results. Proinflammatory fragments described, with appropriate references.
