# Supplementary material for: The Arg/N-Degron Pathway—A Potential Running Back in Fine-Tuning the Inflammatory Response?
Source: Biomolecules. 2020 Jun 14;10(6):903. doi: 10.3390/biom10060903 (PMC7356051; doi:10.3390/biom10060903)
Supplement: Supplementary file 1 [file biomolecules-10-00903-s001.zip › biomolecules-816749-supplementary-2_PK corr.docx]

Supplementary Tables

**Table S1.** Proinflammatory fragments with destabilizing N-terminal residues.

| **Protein** | **Cleavage site** | **C-terminal fragment** |
| --- | --- | --- |
| **Experimentally confirmed N-degron pathway substrates** | | |
| hCASP-1 | 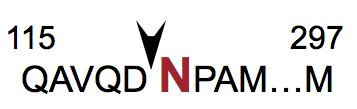 | Proinflammatory fragment, generated by auto-cleavage. Asn-CASP1 is an N-degron pathway substrate. |
| hCASP-4 | 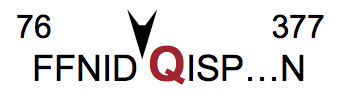 | Proinflammatory fragment, generated by auto-cleavage. Gln-CASP4 is an N-degron pathway substrate. |
| hCASP-5 | 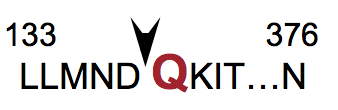 | Proinflammatory fragment, generated by auto-cleavage. Gln-CASP5 is an N-degron pathway substrate. |
| hRAB39a | 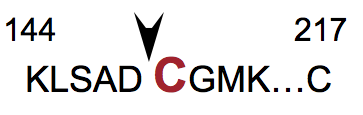 | Proinflammatory fragment, generated by caspase-1 mediated cleavage. Cys-RAB39a is an N-degron pathway substrate. |
| mGRZA | 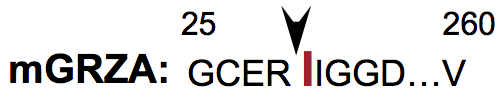 | Proinflammatory fragment, generated by endopeptidase DPP1 mediated cleavage. Ile-GRZA is an N-degron pathway substrate. |
| mGRZM | 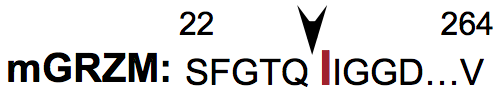 | Proinflammatory fragment, generated by endopeptidase DPP1 mediated cleavage. Ile-GRZM is an N-degron pathway substrate. |
| **Proposed N-degron pathway substrates** | | |
| hIL-18 | 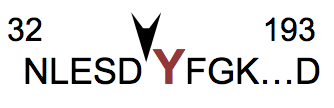 | Proinflammatory fragment, generated by caspase-1, of the cytokine IL-18. Tyr-IL-18 is a likely N-degron pathway substrate. |
| hIL-36β | 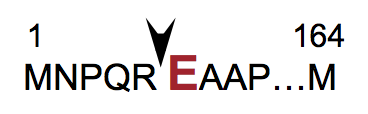 | Proinflammatory fragment, generated by Cathepsin-G or Proteinase-3, of the cytokine IL-36β. Glu-IL-36β is a likely N-degron pathway substrate. |
| hIL36γ | 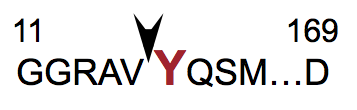 | Proinflammatory fragment, generated by Elastase or Proteinase-3, of the cytokine IL-36γ. Tyr-IL-36γ is a likely N-degron pathway substrate. |
| hCCL3 | 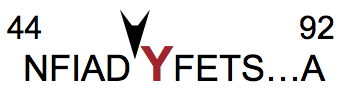 | Proinflammatory fragment, generated by caspase-1, of the chemokine CCL3. Tyr-CCL3 is a likely N-degron pathway substrate. |
| hAtaxin-3 | 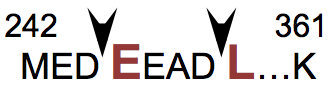 | Proinflammatory fragment, generated by caspase-1, of the protein Ataxin-3. Glu-Ataxin-3 and Leu-Ataxin-3 are likely N-degron pathway substrates. |
| hhnRNPA2 | 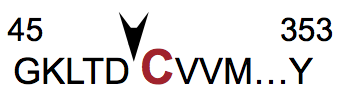 | Proinflammatory fragment, generated by caspase-1, of the protein hnRNPA2. Cys-hnRNPA2 is a likely N-degron pathway substrate. |
| hMatrin-3 | 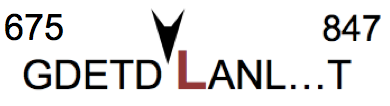 | Proinflammatory fragment, generated by caspase-5, of the protein Matrin-3. Leu-Matrin-3 is a likely N-degron pathway substrate. |
| mGRZK | 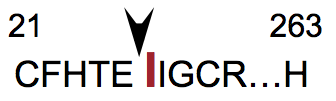 | Proinflammatory fragment, generated by endopeptidase DPP1 mediated cleavage. Ile-GRZK is an N-degron pathway substrate. |

**Table S2: Plasmids used in this study.**

| **Plasmid** | **Description** | **Reference** |
| --- | --- | --- |
| pKP496 | Amp^R^; Neo^R^; pcDNA3.0-based plasmid encoding flag-DHFR-ha Ub-MCS-flag under the control of CMV promoter; MCS has SacII, EcoRI, XhoI, ClaI, and EcoRV unique cloning sites | [1] |
| pKP502 | Amp^R^; Neo^R^; pcDNA3.0-based plasmid encoding flag-DHFR-ha Ub-Asn^120^-hCASP-1-flag under the control of CMV promoter | This study |
| pKP503 | Amp^R^; Neo^R^; pcDNA3.0-based plasmid encoding flag-DHFR-ha Ub- Val^120^-hCASP-1-flag under the control of CMV promoter | This study |
| pKP504 | Amp^R^; Neo^R^; pcDNA3.0-based plasmid encoding flag-DHFR-ha Ub-Gln^81^-hCASP-4-flag under the control of CMV promoter | This study |
| pKP505 | Amp^R^; Neo^R^; pcDNA3.0-based plasmid encoding flag-DHFR-ha Ub- Val^81^-hCASP-4-flag under the control of CMV promoter | This study |
| pKP506 | Amp^R^; Neo^R^; pcDNA3.0-based plasmid encoding flag-DHFR-ha Ub- Gln^138^-hCASP-5-flag under the control of CMV promoter | This study |
| pKP507 | Amp^R^; Neo^R^; pcDNA3.0-based plasmid encoding flag-DHFR-ha Ub- Val^138^-hCASP-5-flag under the control of CMV promoter | This study |
| pKP510 | Amp^R^; Neo^R^; pcDNA3.0-based plasmid encoding flag-DHFR-ha Ub-Cys^149^-hRAB39a-flag under the control of CMV promoter | This study |
| pKP511 | Amp^R^; Neo^R^; pcDNA3.0-based plasmid encoding flag-DHFR-ha Ub- Val^149^-hRAB39a-flag under the control of CMV promoter | This study |
| pDL1 | Amp^R^; Neo^R^; pcDNA3.0-based plasmid encoding flag-DHFR-ha Ub- Ile^29^-mGRZA-flag under the control of CMV promoter | This study |
| pDL2 | Amp^R^; Neo^R^; pcDNA3.0-based plasmid encoding flag-DHFR-ha Ub- Val^29^-mGRZA-flag under the control of CMV promoter | This study |
| pDL7 | Amp^R^; Neo^R^; pcDNA3.0-based plasmid encoding flag-DHFR-ha Ub- Ile^27^-mGRZM-flag under the control of CMV promoter | This study |
| pDL8 | Amp^R^; Neo^R^; pcDNA3.0-based plasmid encoding flag-DHFR-ha Ub- Val^27^-mGRZM-flag under the control of CMV promoter | This study |

**Table S3.** Primers used in this study.

| **Number** | **Sequence, 5’to 3’** |
| --- | --- |
| **1414** | AAAAACCGCGGAGGAAACCCAGCTATGCCCACATCCT |
| **1415** | AAAAACCGCGGAGGAgttCCAGCTATGCCCACATCCT |
| **1416** | TTTTAATCGATATCTTTAAACCACACCACACCAGG |
| **1417** | AAAAACCGCGGAGGACAAATATCCCCCAATAAAAAAGCTC |
| **1418** | AAAAACCGCGGAGGAgttATATCCCCCAATAAAAAAGCTC |
| **1419** | TTTTAATCGATGTCTCTGACCCACAGTTCCCC |
| **1420** | AAAAACCGCGGAGGACAAAAGATCACCAGTGTAAAACCTCTTC |
| **1421** | AAAAACCGCGGAGGAgttAAGATCACCAGTGTAAAACCTCTTC |
| **1422** | TTTTAATCGATGTCTCTGACCCAGAGTTCCCCA |
| **1447** | GGGAATTCCTCGAGATCGATATCGACTACAAAGACGATGACGACAAAGGTTAAT |
| **1448** | CTAGATTAACCTTTGTCGTCATCGTCTTTGTAGTCGATATCGATCTCGAGGAATTCCCGC |
| **1451** | AAAAACCGCGGAGGATGTGGAATGAAGTACATAGAGACCTCAGCC |
| **1452** | AAAAACCGCGGAGGAgttGGAATGAAGTACATAGAGACCTCAGCC |
| **1453** | TTTTAATCGATCATAGCCATACAGAAGCACTCTTTTCTGGGCTT |
| **1964** | AAAAACCGCGGAGGAATCATTGGAGGAGACACGGTTGTTCCTC |
| **1965** | AAAAACCGCGGAGGAgttaagGGAGGAGACACGGTTGTTCCTC |
| **1968** | TTTTTATCGATCACAGAACCCTTCATAATCTTCTTTATCC |
| **1979** | AAAAACCGCGGAGGAATCATTGGGGGTCGAGAGGCAGTCCCGC |
| **1980** | AAAAACCGCGGAGGAgttaagGGGGGTCGAGAGGCAGTCCCGC |
| **1983** | TTTTTATCGATGACCAAAGATTGGGGTGACCAGCGACCAAT |
|  |  |
| **mGAPDH dir** | AGGTCGGTGTGAACGGATTTG |
| **mGAPDH rev** | TGTAGACCATGTAGTTGAGGTCA |
| **mUbr1up** | CCCAGCAGTTCCTGTCTTGT |
| **mUbr1lo** | ATCAGGAGGCACTTTCAGGC |
| **mUbr2up** | AGAGTTTTCAGTCGCAGACCT |
| **mUbr2lo** | TGATCGGGTCCATTCCCTGC |
| **mUbr4up** | GCAGGGAGGGGTACAAGTTC |
| **mUbr4lo** | GGCCTCTAGCCAACCTGAC |
| **mUbr5up** | AGAACCATTACCACCACGGC |
| **mUbr5lo** | CCACCTCAACCTCTTCCACG |

**Table S4.** List of siRNA.

|  | **Sense** | **Anti-sense** |
| --- | --- | --- |
| si-LUC | cuuAcGcuGAGuAcuucGATsT | UCGAAGuACUcAGCGuAAGTsT |
| si-Ubr1 | caGAcuAGGuGcuauuucATsT | UGAAAuAGcACCuAGUCUGTsT |
| si-Ubr2 | ggcGAGAGAuGuucGAcAATsT | UUGUCGAAcAUCUCUCGCCTsT |
| si-Ubr4 | ggAcAuGAccAcAgGuAcATsT | UGuACCUGUGGUcAUGUCCTsT |
| si-Ubr5 | ugAuAAGGAuGGAacAAAATsT | UUUUGUUCcAUCCUuAUcATsT |

Uppercase letters: ribonucleotides; Lowercase letters: 2’-O-Methyl nucleotides; s: phosphorothioate.

Supplementary Methods

**Description of plasmids used in this study**

**hCASP-1.** The human Caspase-1 ORF was amplified using cDNA from OpenBiosystems (5583549) and primers 1414 and 1416 for Asn^120^-hCASP-1 or 1415 and 1416 for Val^120^-hCASP-1. The resulting fragments were cut with SacII and ClaI and cloned into SacII/ ClaI-cut pKP496, generating the plasmids pKP502 and pKP503, respectively.

**hCASP-4.** The human Caspase-4 ORF was amplified using cDNA from OpenBiosystems (6276763) and primers 1417 and 1419 for Gln^81^-hCASP-4 or 1418 and 1419 for Val^81^-hCASP-4. The resulting fragments were cut with SacII and ClaI and cloned into SacII/ ClaI-cut pKP496, generating the plasmids pKP504 and pKP505, respectively.

**hCASP-5.** The human Caspase-5 ORF was amplified using cDNA from OpenBiosystems (30915395) and primers 1,420 and 1,422 for Gln^138^-hCASP-5 or 1421 and 1422 for Val^138^-hCASP-5. The resulting fragments were cut with SacII and ClaI and cloned into SacII/ ClaI-cut pKP496, generating the plasmids pKP506 and pKP507, respectively.

**hRab39a.** The human Rab39a ORF was amplified using cDNA from OpenBiosystems (5583549) and primers 1451 and 1453 for Cys^149^-hRab39a or 1452 and 1453 for Val^149^-hRab39a. The resulting fragments were cut with SacII and ClaI and cloned into SacII/ ClaI-cut pKP496, generating the plasmids pKP510 and pKP511, respectively.

**mGRZA.** The mouse GrzA ORF was amplified using a cDNA library derived from the C57Bl/6J mouse RNA and primers 1964 and 1968 for Ile^29^-mGRZA or 1965 and 1968 for Val^29^-mGRZA. The resulting fragments were cut with SacII and ClaI and cloned into SacII/ ClaI-cut pKP496, generating the plasmids pDL1 and pDL2, respectively.

**mGRZM.** The mouse GrzM ORF was amplified using cDNA from C57Bl/6J mouse and primers 1979 and 1983 for Ile^27^-mGRZM or 1980 and 1983 for Val^27^-mGRZM. The resulting fragments were cut with SacII and ClaI and cloned into SacII/ ClaI-cut pKP496, generating the plasmids pDL7 and pDL8, respectively.

Supplementary results

**Pro-inflammatory fragments described.**

The pro-inflammatory fragments included in this study are detailed below. These fragments are produced by caspases or other endopeptidases (see Supplementary table 1) and have been shown, in the present work, to be actual or potential short-lived substrates of the Arg/N-degron pathway (Fig. 2 and Supplementary Figs. S2-4).

**^120^Asn-CASP1**. Originally discovered for its role in the secretion of IL1β [2], caspase-1 is the most studied and best characterized caspase, and is known to cleave many pro-inflammatory and pro-pyroptosis fragments, including other members of the IL1 family [3–8]. Caspase-1 is initially translated as a zymogen and requires interaction with the inflammasome for dimerization and activation. Self-cleavage of caspase-1 generates a more stable active form of the proteolytic enzyme and even serves to terminate inflammasome activity [9]. In all cases, activation of caspase-1 results in cleavage of pro-inflammatory peptides which lead to IL1β secretion and pyroptosis of the infected cells. Pyroptosis is a unique cell death mechanism where rapid plasma-membrane rupture releases pro-inflammatory cellular contents into the extracellular space. It is caspase-1 dependant, and occurs after canonical or non-canonical inflammasome activation [10]. Caspase-1 is also capable of activating the NF-kB pathway through interaction of its CARD domain with RIP2 [11].

**^81^Gln-CASP4**. Caspase-4 is an inflammation initiator caspase with 53% homology to caspase-1. Similarly, caspase-4 is initially produced as a zymogen and requires dimerization and interdomain processing for activation [12]. Caspase-4 is required for the activation of caspase-1 through non-canonical inflammasomes, which involves detection and direct binding with intracellular LPS [13]. It is also an essential effector of NLRP3 inflammasome dependent IL-1β and IL-18 secretion in response to non-canonical activators, such as UVB radiation and cholera enterotoxin subunit B, which leads to pyroptosis through Gasdermin D cleavage and activation [14–16]. It does not process IL-1β directly.

**^138^Gln-CASP5**. Human caspase-5, with caspase-4, have been suggested to originate from a duplication of the murine caspase-11. Caspase-5 was originally cloned from human THP-1 cells and placenta tissue and was named ICErelIII and TY, respectively [17]. It shares 51% sequence homology with caspase-1 and 74% with caspase-4. However, contrarily to caspase-4, the expression of caspase-5 is restricted to the placenta, lung, liver, spleen, small intestine, colon, and peripheral blood lymphocytes [18]. The roles, functions and activation mechanisms of caspase-5 are similar to caspase-4, and it is involved in many skin conditions such as psoriasis and lupus through activation by the NLRP1 inflammasome [19].

**^149^Cys-RAB39a**. Rab39a is a member of the Rab-GTPase family of proteins, which are responsible for vesicle trafficking in pathways such as secretion and endocytosis [20]. Rab39a is a necessary caspase-1 binding partner for the secretion of IL1β. Cleavage of Rab39a by caspase-1 at the conserved cleavage site allows the release of the active form of IL1β from cells.

**^29^Ile-GRZA**. Granzyme A has been demonstrated to process IL-1b into its active form, in a caspase-1 independent manner, in the context of a *Pasteurella multocida* infection [21]. Both mouse and human granzyme A could induce pro-inflammatory cytokine secretion (IL-1b, IL-6, IL-8 an TNFa) from peripheral blood monocytes, once internalized into the cells [22]. Additionally, granzyme A is capable of activating TLR9 in the endosomes of plasmacytoid dendritic cells, switching on their maturation program and inducing type I interferon production [23].

**^26^Ile-GRZK**. In the context of LCMV infection, granzyme K was shown to induce active IL-1b release from macrophages, and this is specific to granzyme K as neither granzyme A or B were able to induce cytokine production after LCMV challenge [24].

**^27^Ile-GRZM**. A role for granzyme M in the secretion of proinflammatory cytokines such as IL-1a, IL-1b, TNFa and IFNg was discovered when mice KO for this granzyme failed to secrete these cytokines in comparable levels to controls following LPS stimulation [25]. However, the mechanism of how granzyme M increases the signaling cascade and cytokine production downstream of the LPS/TLR4 activation remains unknown. Granzyme M is also required for maximal secretion of MIP1a following *Listeria monocytogenes* infection [26].

**^37^Tyr-IL-18, ^6^Glu-IL-36**β **and ^16^Tyr-IL-36γ**. IL-18 and IL-36 belong to the IL-1 family of cytokines, which is closely linked to inflammation and non-specific response to infection and foreign antigens (reviewed in [27]). Both IL-18 and IL-36 are pro-inflammatory cytokines, and are involved in INF-γ and TNFα production, chemokine secretion, expression of vascular cell adhesion molecules and have a role in auto-immune skin conditions such as psoriasis ([28,29] and references therein). proIL-18 is processed by caspase-1 after Asp^36^ into its active form, however processing of this cytokine can also occur by mast cell chymase (after Phe^57^), or by elastase (after Val^98^, Leu^143^ and Val^159^) [30]. Interestingly, all these cuts by proteases generate destabilising N-termini, suggesting that this processing destines the IL-18 peptides for degradation, a hypothesis that is corroborated by the loss of activity post-processing by elastase. IL-36 is not cleaved by caspase-1, but has multiple cleavage sites for neutrophil-derived proteases such as cathepsin-G, elastase and proteinase-3 [31]. The ^37^Tyr-IL-18, ^6^Glu-IL-36β and ^16^Tyr-IL-36γ fragments are likely Arg/N-degron pathway substrates.

**^49^Tyr-CCL3**. Macrophage inflammatory protein 1a, (MIP-1a) also known as CCL3, is a pro-inflammatory chemoattractant for monocyte-lineage cells and lymphocytes into inflammatory tissue [32]. CCL3 has a caspase-1 cleavage site at Asp^48^, revealing a destabilising residue at position 49, and making this newly formed peptide a likely Arg/N-degron pathway substrate. However, it is unclear whether or not cleavage by caspase-1 is necessary for the pro-inflammatory action of CCL3. Mutational analysis of the amino acids composing CCL3 revealed that the Asp^48^ is critical for aggregation of MIP1α [33], and that aggregation is necessary for the chemokine activity of this protein [34]. This implies the importance of the caspase-1 cleavage site for optimal activity of CCL3.

**^245^Glu-Ataxin-3 and ^249^Leu-Ataxin-3**. Polyglutamine tract protein defective in spinocerebrellar ataxia type 3 (Ataxin-3) is a deubiquitinating enzyme involved in protein homeostasis maintenance, transcription, cytoskeleton regulation, myogenesis and degradation of misfolded chaperone substrates. It is processed by caspase-1 at the Asp^244^ and Asp^248^ sites, generating two likely Arg/N-degron pathway substrates, although this remains to be experimentally proven. Cleavage by caspase-1 causes the protein fragments to aggregate, which could lead to neurodegeneration, inflammation and apoptosis [35,36].

**^50^Cys-hnRNPA2**. The Heterogeneous nuclear ribonucleoprotein 2 (hnRNPA2) associates with nascent pre-mRNAs, packaging them into hnRNP particles. Packaging plays a role in various processes such as transcription, pre-mRNA processing, RNA nuclear export, subcellular location, mRNA translation and stability of mature mRNAs. hnRNPA2 is crucial for embryonic development [37], however, direct roles in inflammation have yet to be uncovered. hnRNPA2 is cleaved by caspase-1 at the Asp^49^ site, and is a likely Arg/N-degron pathway substrate.

**^681^Leu-Matrin-3**. Matrin 3 is a Ca2+/calmodulin-binding protein cleaved by caspase-5 at the Asp680 position [38], and is a likely Arg/N-degron pathway substrate. Cleavage by caspases modulates the activity of the protein. Matrin-3 has recently been involved in the DNA-mediated innate immune response, along with long-noncoding RNAs HEXIM1 and NEAT1 [39].

References

1. Piatkov, K.I.; Brower, C.S.; Varshavsky, A. The N-end rule pathway counteracts cell death by destroying proapoptotic protein fragments. *Proc. Natl. Acad. Sci.* **2012**, *109*, E1839–E1847, doi:10.1073/pnas.1207786109.
2. Cerretti, D.; Kozlosky, C.; Mosley, B.; Nelson, N.; Van Ness, K.; Greenstreet, T.; March, C.; Kronheim; Druck, T.; Cannizzaro, L.; et al. Molecular cloning of the interleukin-1 beta converting enzyme. *Sci.* **1992**, *256*, 97–100, doi:10.1126/science.1373520.
3. Afonina, I.S.; Müller, C.; Martin, S.J.; Beyaert, R. Proteolytic Processing of Interleukin-1 Family Cytokines: Variations on a Common Theme. *Immun.* **2015**, *42*, 991–1004, doi:10.1016/j.immuni.2015.06.003.
4. Dénes, A.; Lopez-Castejon, G.; Brough, D. Caspase-1: is IL-1 just the tip of the ICEberg? *Cell Death Dis.* **2012**, *3*, e338, doi:10.1038/cddis.2012.86.
5. Lamkanfi, M.; Kanneganti, T.-D.; Van Damme, P.; Berghe, T.V.; Vanoverberghe, I.; Vandekerckhove, J.; Vandenabeele, P.; Gevaert, K.; Núñez, G. Targeted peptidecentric proteomics reveals caspase-7 as a substrate of the caspase-1 inflammasomes. *Mol. Cell. Proteom.* **2008**, *7*, 2350–63, doi:10.1074/mcp.M800132-MCP200.
6. Shao, W.; Yeretssian, G.; Doiron, K.; Hussain, S.N.; Saleh, M. The Caspase-1 Digestome Identifies the Glycolysis Pathway as a Target during Infection and Septic Shock. *J. Boil. Chem.* **2007**, *282*, 36321–36329, doi:10.1074/jbc.m708182200.
7. Shen, J.; Yin, Y.; Mai, J.; Xiong, X.; Pansuria, M.; Liu, J.; Maley, E.; Saqib, N.U.; Wang, H.; Yang, X. Caspase-1 recognizes extended cleavage sites in its natural substrates. *Atheroscler.* **2009**, *210*, 422–9, doi:10.1016/j.atherosclerosis.2009.12.017.
8. Wang, L.; Fu, H.; Nanayakkara, G.; Li, Y.; Shao, Y.; Johnson, C.; Cheng, J.; Yang, W.Y.; Yang, F.; Lavallee, M.; et al. Novel extracellular and nuclear caspase-1 and inflammasomes propagate inflammation and regulate gene expression: a comprehensive database mining study. *J. Hematol. Oncol.* **2016**, *9*, 122, doi:10.1186/s13045-016-0351-5.
9. Boucher, D.; Monteleone, M.; Coll, R.C.; Chen, K.W.; Ross, C.M.; Teo, J.L.; Gomez, G.A.; Holley, C.L.; Bierschenk, D.; Stacey, K.J.; et al. Caspase-1 self-cleavage is an intrinsic mechanism to terminate inflammasome activity. *J. Exp. Med.* **2018**, *215*, 827–840, doi:10.1084/jem.20172222.
10. Bergsbaken, T.; Fink, S.L.; Cookson, B.T. Pyroptosis: host cell death and inflammation. *Nat. Rev. Genet.* **2009**, *7*, 99–109, doi:10.1038/nrmicro2070.
11. Lamkanfi, M.; Kalai, M.; Saelens, X.; Declercq, W.; Vandenabeele, P. Caspase-1 Activates Nuclear Factor of the κ-Enhancer in B Cells Independently of Its Enzymatic Activity. *J. Boil. Chem.* **2004**, *279*, 24785–24793, doi:10.1074/jbc.m400985200.
12. Karki, P.; Dahal, G.R.; Park, I.-S. Both dimerization and interdomain processing are essential for caspase-4 activation. *Biochem. Biophys. Res. Commun.* **2007**, *356*, 1056–1061, doi:10.1016/j.bbrc.2007.03.102.
13. Shi, J.; Zhao, Y.; Wang, Y.; Gao, W.; Ding, J.; Li, P.; Hu, L.; Shao, F. Inflammatory caspases are innate immune receptors for intracellular LPS. *Nat.* **2014**, *514*, 187–192, doi:10.1038/nature13683.
14. Sollberger, G.; Strittmatter, G.E.; Kistowska, M.; French, L.; Beer, H.-D. Caspase-4 Is Required for Activation of Inflammasomes. *J. Immunol.* **2012**, *188*, 1992–2000, doi:10.4049/jimmunol.1101620.
15. Knodler, L. A., S. M. Crowley, H. P. Sham, H. Yang, M. Wrande, C. Ma, R. K. Ernst, O. Steele-Mortimer, J. Celli, and B. A. Vallance. "Noncanonical Inflammasome Activation of Caspase-4/Caspase-11 Mediates Epithelial Defenses against Enteric Bacterial Pathogens." Cell Host Microbe 16, no. 2 (2014): 249-56.
16. Viganò, E.; Diamond, C.; Spreafico, R.; Balachander, A.; Sobota, R.M.; Mortellaro, A. Human caspase-4 and caspase-5 regulate the one-step non-canonical inflammasome activation in monocytes. *Nat. Commun.* **2015**, *6*, 8761, doi:10.1038/ncomms9761.
17. Munday, N.A.; Vaillancourt, J.P.; Ali, A.; Casano, F.J.; Miller, D.K.; Molineaux, S.M.; Yamin, T.-T.; Yu, V.L.; Nicholson, D.W. Molecular Cloning and Pro-apoptotic Activity of ICErelII and ICErelIII, Members of the ICE/CED-3 Family of Cysteine Proteases. *J. Boil. Chem.* **1995**, *270*, 15870–15876, doi:10.1074/jbc.270.26.15870.
18. Lin, X.Y.; Choi, M.S.K.; Porter, A.G. Expression Analysis of the Human Caspase-1 Subfamily Reveals Specific Regulation of theCASP5Gene by Lipopolysaccharide and Interferon-γ. *J. Boil. Chem.* **2000**, *275*, 39920–39926, doi:10.1074/jbc.m007255200.
19. Zwicker, S.; Hattinger, E.; Bureik, D.; Batycka-Baran, A.; Schmidt, A.; Gerber, P.-A.; Rothenfusser, S.; Gilliet, M.; Ruzicka, T.; Wolf, R. Th17 micro-milieu regulates NLRP1-dependent caspase-5 activity in skin autoinflammation. *PLOS ONE* **2017**, *12*, e0175153, doi:10.1371/journal.pone.0175153.
20. Zerial, M.; McBride, H.M. Rab proteins as membrane organizers. *Nat. Rev. Mol. Cell Boil.* **2001**, *2*, 107–117, doi:10.1038/35052055.
21. Hildebrand, D.; Bode, K.A.; Ries, D.; Cerny, D.; Waldhuber, A.; Römmler, F.; Strack, J.; Korten, S.; Orth, J.H.C.; Miethke, T.; et al. Granzyme A Produces Bioactive IL-1β through a Nonapoptotic Inflammasome-Independent Pathway. *Cell Rep.* **2014**, *9*, 910–917, doi:10.1016/j.celrep.2014.10.003.
22. Metkar, S.S.; Menaa, C.; Pardo, J.; Wang, B.; Wallich, R.; Freudenberg, M.; Kim, S.; Raja, S.M.; Shi, L.; Simon, M.M.; et al. Human and Mouse Granzyme A Induce a Proinflammatory Cytokine Response. *Immun.* **2008**, *29*, 720–733, doi:10.1016/j.immuni.2008.08.014.
23. Shimizu, K.; Yamasaki, S.; Sakurai, M.; Yumoto, N.; Ikeda, M.; Mishima-Tsumagari, C.; Kukimoto-Niino, M.; Watanabe, T.; Kawamura, M.; Shirouzu, M.; et al. Granzyme A Stimulates pDCs to Promote Adaptive Immunity via Induction of Type I IFN. *Front. Immunol.* **2019**, *10*, 1450, doi:10.3389/fimmu.2019.01450.
24. Joeckel, L.T.; Wallich, R.; Martin, P.; Sanchez, D.; Weber, F.C.; Martin, S.F.; Borner, C.; Pardo, J.; Froelich, C.; Simon, M.M. Mouse granzyme K has pro-inflammatory potential. *Cell Death Differ.* **2011**, *18*, 1112–1119, doi:10.1038/cdd.2011.5.
25. Anthony, D.A.; Andrews, D.; Chow, M.; Watt, S.V.; House, C.; Akira, S.; Bird, P.; Trapani, J.A.; Smyth, M.J. A Role for Granzyme M in TLR4-Driven Inflammation and Endotoxicosis. *J. Immunol.* **2010**, *185*, 1794–1803, doi:10.4049/jimmunol.1000430.
26. Baschuk, N.; Wang, N.; Watt, S.V.; Halse, H.; House, C.; Bird, P.; Strugnell, R.A.; Trapani, J.A.; Smyth, M.J.; Andrews, D. NK cell intrinsic regulation of MIP-1α by granzyme M. *Cell Death Dis.* **2014**, *5*, e1115, doi:10.1038/cddis.2014.74.
27. Dinarello, C.A. Overview of the IL-1 family in innate inflammation and acquired immunity. *Immunol. Rev.* **2017**, *281*, 8–27, doi:10.1111/imr.12621.
28. Gracie, J.A.; Robertson, S.E.; McInnes, I. Interleukin-*J. Leukoc. Boil.* **2003**, *73*, 213–224, doi:10.1189/jlb.0602313.
29. Gresnigt, M.; Van De Veerdonk, F.L. Biology of IL-36 cytokines and their role in disease. *Semin. Immunol.* **2013**, *25*, 458–465, doi:10.1016/j.smim.2013.11.003.
30. Robertson, S.E.; Young, J.D.; Kitson, S.; Pitt, A.R.; Evans, J.; Roes, J.; Karaoglu, D.; Santora, L.; Ghayur, T.; Liew, F.Y.; et al. Expression and alternative processing of IL-18 inhuman neutrophils. *Eur. J. Immunol.* **2006**, *36*, 722–731, doi:10.1002/eji.200535402.
31. Henry, C.M.; Sullivan, G.P.; Clancy, D.; Afonina, I.S.; Kulms, D.; Martin, S.J. Neutrophil-Derived Proteases Escalate Inflammation through Activation of IL-36 Family Cytokines. *Cell Rep.* **2016**, *14*, 708–722, doi:10.1016/j.celrep.2015.12.072.
32. Cook, D.N. The role of MIP-1 alpha in inflammation and hematopoiesis. *J. Leukoc. Boil.* 1996, *59*, 61–6.
33. Czaplewski, L.G.; McKeating, J.; Craven, C.J.; Higgins, L.D.; Appay, V.; Brown, A.; Dudgeon, T.; A Howard, L.; Meyers, T.; Owen, J.; et al. Identification of amino acid residues critical for aggregation of human CC chemokines macrophage inflammatory protein (MIP)-1alpha, MIP-1beta, and RANTES. Characterization of active disaggregated chemokine variants. *J. Boil. Chem.* 1999, *274*, 16077–84.
34. Ren, M.; Guo, Q.; Guo, L.; Lenz, M.; Qian, F.; Koenen, R.R.; Xu, H.; Schilling, A.B.; Weber, C.; Ye, R.D.; et al. Polymerization of MIP-1 chemokine (CCL3 and CCL4) and clearance of MIP-1 by insulin-degrading enzyme. *EMBO J.* **2010**, *29*, 3952–3966, doi:10.1038/emboj.2010.256.
35. Wellington, C.L.; Ellerby, L.M.; Hackam, A.S.; Margolis, R.L.; Trifiro, M.A.; Singaraja, R.R.; McCutcheon, K.; Salvesen, G.S.; Propp, S.S.; Bromm, M.; et al. Caspase cleavage of gene products associated with triplet expansion disorders generates truncated fragments containing the polyglutamine tract. *J. Boil. Chem.* **1998**, *273*, 9158–9167, doi:10.1074/jbc.273.15.9158.
36. Jadhav, S.; Žilka, N.; Novak, M. Protein Truncation as a Common Denominator of Human Neurodegenerative Foldopathies. *Mol. Neurobiol.* **2013**, *48*, 516–532, doi:10.1007/s12035-013-8440-8.
37. Kwon, J.; Jo, Y.-J.; Namgoong, S.; Kim, N.-H. Functional roles of hnRNPA2/B1 regulated by METTL3 in mammalian embryonic development. *Sci. Rep.* **2019**, *9*, 8640, doi:10.1038/s41598-019-44714-1.
38. Valencia, C.A.; Ju, W.; Liu, R. Matrin 3 is a Ca2+/calmodulin-binding protein cleaved by caspases. *Biochem. Biophys. Res. Commun.* **2007**, *361*, 281–286, doi:10.1016/j.bbrc.2007.06.156.
39. Morchikh, M.; Cribier, A.; Raffel, R.; Amraoui, S.; Cau, J.; Severac, D.; Dubois, E.; Schwartz, O.; Bennasser, Y.; Benkirane, M. HEXIM1 and NEAT1 Long Non-coding RNA Form a Multi-subunit Complex that Regulates DNA-Mediated Innate Immune Response. *Mol. Cell* **2017**, *67*, 387–399.e5, doi:10.1016/j.molcel.2017.06.020.

| 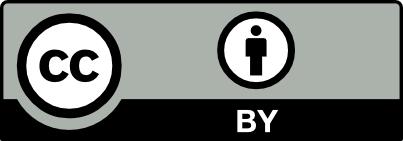 | © 2020 by the authors. Submitted for possible open access publication under the terms and conditions of the Creative Commons Attribution (CC BY) license (http://creativecommons.org/licenses/by/4.0/). |
| --- | --- |
