# Supplementary figures and images for: The Arg/N-Degron Pathway—A Potential Running Back in Fine-Tuning the Inflammatory Response?
Source: Biomolecules. 2020 Jun 14;10(6):903. doi: 10.3390/biom10060903 (PMC7356051; doi:10.3390/biom10060903)

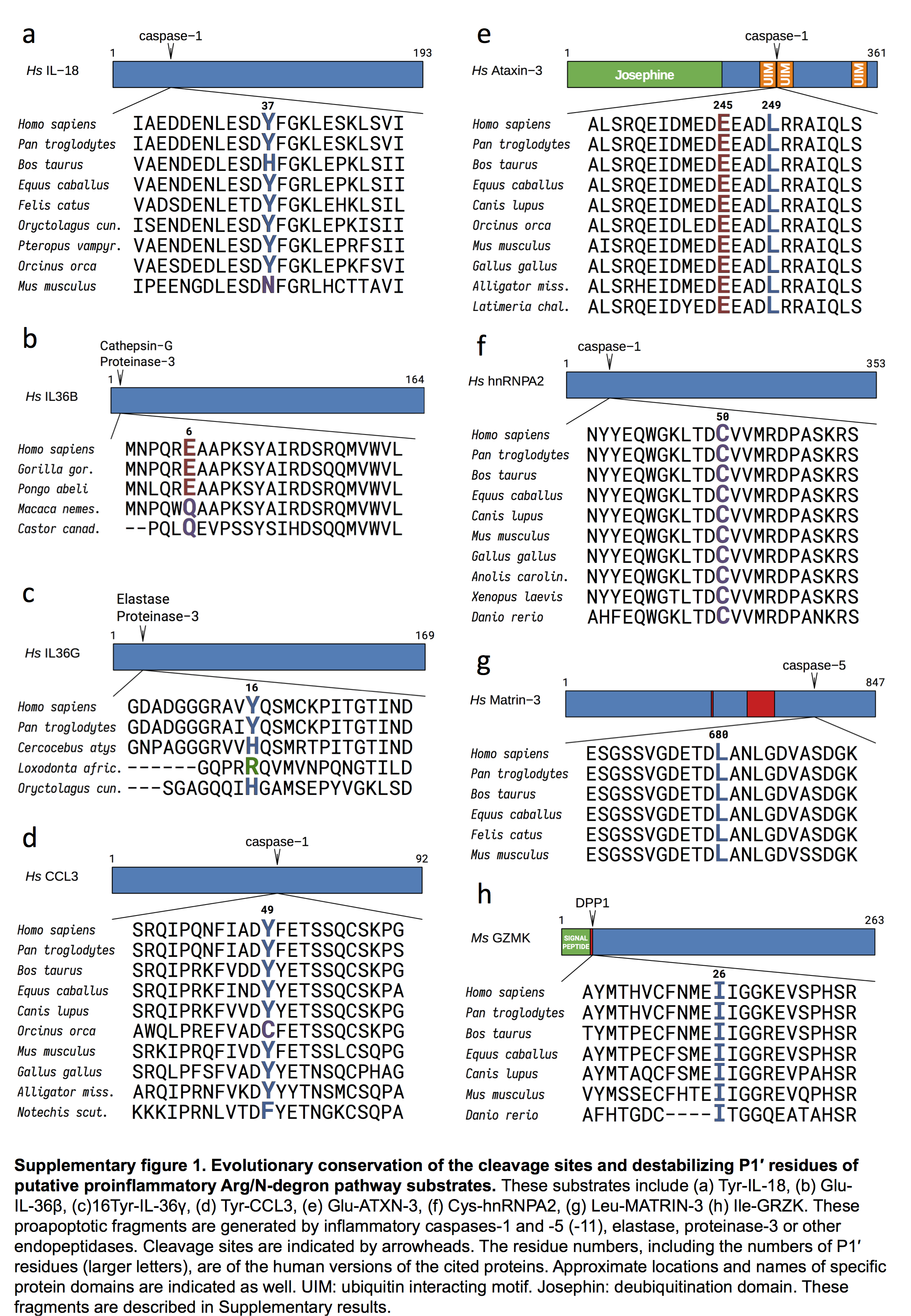

Supplement: Supplementary file 1 [file biomolecules-10-00903-s001.zip › Supp Figure NDP Inflammation.tiff]
